# The effects and interaction of soybean maturity gene alleles controlling flowering time, maturity, and adaptation in tropical environments

**DOI:** 10.1186/s12870-020-2276-y

**Published:** 2020-02-07

**Authors:** Carrie Miranda, Andrew Scaboo, Elroy Cober, Nicholas Denwar, Kristin Bilyeu

**Affiliations:** 1 USDA/ARS Plant Genetics Research Unit, 110 Waters Hall, University of Missouri, Columbia, MO 65211 USA; 2 Division of Plant Sciences, 110 Waters Hall, University of Missouri, Columbia, MO 65211 USA; 3 Ottawa Research and Development Centre, Agriculture and Agri-Food Canada, 960 Carling Ave., Ottawa, Ontario K1A 0C6 Canada; 4 CSIR-Savanna Agricultural Research Institute, P. O. Box 52, Tamale, Ghana

**Keywords:** Soybean, Long juvenile trait, Africa, Low latitude, Maturity, Tropical environment, Terminal stem growth, Ghana

## Abstract

**Background:**

Soybean is native to the temperate zones of East Asia. Poor yields of soybean in West African countries may be partially attributed to inadequate adaptation of soybean to tropical environments. Adaptation will require knowledge of the effects of allelic combinations of major maturity genes (*E1*, *E2*, and *E3*) and stem architecture. The long juvenile trait (*J*) influences soybean flowering time in short, ~ 12 h days, which characterize tropical latitudes. Soybean plant architecture includes determinate or indeterminate stem phenotypes controlled by the *Dt1* gene. Understanding the influence of these genetic components on plant development and adaptation is key to optimize phenology and improve soybean yield potential in tropical environments.

**Results:**

Soybean lines from five recombinant inbred populations were developed that varied in their combinations of targeted genes. The soybean lines were field tested in multiple environments and characterized for days to flowering (DTF), days to maturity (DTM), and plant height in locations throughout northern Ghana, and allelic combinations were determined for each line for associating genotype with phenotype. The results revealed significant differences based on genotype for DTF and DTM and allowed the comparison of different variant alleles of those genes. The mutant alleles of *J* and *E1* had significant impact on DTF and DTM, and alleles of those genes interacted with each other for DTF but not DTM. The *Dt1* gene significantly influenced plant height but not DTF or DTM.

**Conclusions:**

This research identified major and minor effect alleles of soybean genes that can be combined to control DTF, DTM, and plant height in short day tropical environments in Ghana. These phenotypes contribute to adaptation to a low latitude environment that can be optimized in a soybean breeding program with targeted selection of desired allele combinations. The knowledge of the genetic control of these traits will enhance molecular breeding to produce optimally adapted soybean varieties targeted to tropical environments.

## Background

Demand for soybean is increasing throughout Africa both for livestock feed and as a protein source to ameliorate malnutrition [1, 2], but sub-Saharan African soybean yields are lower than their potential [1, 3–7]. It is important to ensure the genetic background of tropical soybean grown in West Africa is adapted to compensate for environmental influences such as poor soils or diseases that are difficult or costly to control. Understanding the genetic mechanisms behind agronomic traits such as days to flower and days to maturity will allow soybean breeders to optimize the varieties they release to protect yield potentials, as photoperiod response is the most important trait influencing soybean adaptation in a tropical environment [8].

Soybean was domesticated ~ 5000 years ago in northern China at approximately 35°N latitude [9, 10]. This latitude is characterized by long days > 13 h during the growing season. Soybean is a short day, photoperiod sensitive plant, and flowering is induced by short day length [11–14]. When soybean is grown in a 12 h or less day length, it receives the cue to start flowering immediately upon emergence, making it difficult to adapt to latitudes below 20° [15–19]. This early flowering results in a short stature plant that matures prematurely and leads to reduced yields [20].

In temperate climates, genes controlling growth and maturity are understood. *E1, E2,* and *E3* maturity genes delay flowering when functional and promote early flowering when recessive [21–31]. Plant height is influenced by terminal stem growth determination and impacts node and pod production and yield [32]. Indeterminate plants continue main stem growth and node production after flowering, while determinate plants terminate main stem growth shortly after flowering. Both maturity genes and architecture genes influence plant height [32]. The major gene for plant architecture is *Dt1*, and four independent *dt1* missense alleles of the gene produce determinate plant architecture [22, 33, 34].

It was discovered that it was possible to expand soybean production to ~ 20° by differing use of alleles of the *E* genes, although it did not allow for production to reach subtropical latitudes that were less than 20° [35, 36]. A trait was discovered, named the long juvenile trait, in plant introduction (PI) 159925 from Peru which did allow extended vegetative growth in short day environments [16, 18]. This phenotype was observed again in Brazil through natural variation of the cultivar Paraná which was then named Paranagoiana [37]. At this time, separate names were assigned for two characterized sources of the long juvenile trait: *J* from PI 159925 and *E6* in Paranagoiana, where the recessive allele of each gene conditioned the expression of the long juvenile trait [18, 37]. The long juvenile trait allowed Brazil to expand its soybean production to the low latitude Matto Grosso region [13]. Numerous studies suggested that in certain backgrounds the long juvenile trait was under the control of a single gene [13, 18]. However, delayed flowering was shown in a 1:15 segregation ratio in other studies [36, 38] suggesting that another gene was able to influence the long juvenile phenotype.

The genetic mechanism behind the long juvenile trait in PI 159925 was only discovered recently [39, 40]. A single gene controls the long juvenile trait in PI 159925 and was determined to be the *Arabidopsis* flowering gene ortholog *ELF3* Glyma.04G050200, Wm82.a2.v1 [39, 40] that contained a single nucleotide deletion causing a frameshift mutation in the 4th exon named *j-1* [39]; however, that causative polymorphism was not discovered in the coding sequence of the *ELF3* gene in Paranagoiana. Genetic mapping data has demonstrated that *E6* is also located on chromosome 4 and may be either tightly linked or an undiscovered mutation in *ELF3* from Paranagoiana [41], so herein this allele is referred to as *j-x*. The long juvenile trait may be influenced by multiple genes besides *ELF3,* which are still yet to be confirmed [36, 38]. The interaction of *E1* and the long juvenile trait is only beginning to be understood [39].

Due to the recent cloning of *ELF3* there is a gap in knowledge of the interaction of the long juvenile trait and the *E* maturity genes, particularly in environments with short photoperiods. In addition, molecular breeding in West Africa was recently introduced, and the results of this study enable utilization of molecular tools to make significant improvements for breeding. The objective of this research was to understand the influence of the *E* maturity genes and alleles of the long juvenile trait on days to flower and days to maturity in a West African environment. To do this, five recombinant inbred line (RIL) populations were created that were segregating for the maturity genes and alleles of interest: *E1*/*e1-as*, *E2*/*e2*, *E3*/*e3*, the *j-1* and *j-x* alleles of different sources of the long juvenile trait *J*, and *Dt1/dt1*. These lines were then grown for 2 years in northern Ghana, ~ 9° N latitude, and evaluated for days to flower, maturity, and plant height.

## Results

### Development of recombinant inbred lines (RILs) and characterization of genotypes to test allele combinations for flowering, maturity, and plant architecture in tropical environments

Maximizing soybean yield potential requires optimizing adaptation for plant development in the targeted environments. Utilizing seven parents with contrasting alleles of the major soybean maturity genes, the plant architecture gene for stem termination, and the long juvenile trait, five RIL populations were developed to determine the effect of those allele combinations on phenology and agronomic phenotypes in tropical field environments of northern Ghana (Table 1 and Table 2, Additional file 1). The F_2_ plants in the Jake-15, Jake-Pa, and X97–15 populations were evaluated for the long juvenile trait in a tropical field environment at a commercial winter nursery in Costa Rica at ~11^o^ N latitude, and about 25% of the Jake-15 and Jake-Pa plants that exhibited long juvenile characteristics were selected for advancement. Cloning of the soybean *J* gene for the long juvenile trait on chromosome 04 enabled the development of molecular marker assays to detect alleles of *j-1* from PI 159925. *j-x* from Paranagoiana (PI 628880) was also tracked with a molecular marker assay that assessed the ability to amplify a genomic region that encompassed the last intron and exon of the *ELF3* gene. All of the phenotypically selected long juvenile F_2_ plants in the Jake-15 and Jake-Pa populations contained homozygous *j-1* or *j-x* alleles.

**Table 1:** Description of soybean parent genotypes, days to flower (DTF) and days to maturity (DTM). Data collected over 2 years in northern Ghana

Cultivar	*ELF3*	*E1*	*E2*	*E3*	*Dt1*	DTF	DTM
X97–0101 (X97)	*J*	*e1-as*	*E2*	*E3*	*Dt1*	29.9	92.3
534545	*J*	*e1-as*	*E2*	*E3*	*Dt1*	29.3	87.2
Jake	*J*	*E1*	*E2*	*E3*	*dt1 (R166W)*	31.8	92.8
PI 159925	*j-1*	*E1*	*E2*	*E3*	*dt1 (R166W)*	44.0	111.2
Paranagoiana	*j-x*	*E1*	*E2*	*E3*	*dt1 (R166W)*	48.0	113.6
X5683-1-18 (Can X)	*j-x*	*E1*	*e2*	*e3*	*Dt1*	39.4	105.1
Jenguma	^a^	*E1*	*E2*	*E3*	*dt1 (P113L)*	44.7	115.9

^a^Genetic source of long juvenile trait undetermined

**Table 2:** Alleles segregating and number of recombinant inbred soybean lines (RILs) in five populations

Population	Parent 1 (*J*)	Parent 2 (*j*)	*LJ*	*E1*	*E2*	*E3*	*Dt1*	2016 Plant Generation	2016 RILs	2017 RILs
Jake-15	Jake	PI 159925	*J/j-1*	*E1*	*E2*	*E3*	*dt1*	F4:6	20	9
Jake-Pa	Jake	Paranagoiana	*J/j-x*	*E1*	*E2*	*E3*	*dt1*	F4:6	18	14
X97–15	X97	PI 159925	*J/j-1*	*E1/e1-as*	*E2*	*E3*	*Dt1/dt1*	F4:6	47	5
X97-Jen	X97	Jenguma	^a^	*E1/e1-as*	*E2*	*E3*	*Dt1/dt1*	F3:5	60	41
534-Can	534545	X5683-1-18 (Can X)	*J/j-x*	*E1/e1-as*	*E2/e2*	*E3/e3*	*Dt1*	F4:6	47	33

^a^Genetic source of long juvenile trait undetermined

The F_2_ plants in the X97–15 population contained a broader spectrum of plant development phenotypes and were thus advanced without selection for the long juvenile trait, as were the remaining populations. Four of the five populations utilized a variant *j* allele, but the genetic mechanism for the long juvenile trait in the Ghanaian variety ‘Jenguma’ is not known (Table 1). We targeted 100 RILs for each of the unselected populations. However, due to a variety of factors including population development in a tropical environment, there was a substantial reduction in the number of RILs that produced enough seed for the experiments (Table 2). The entire set of RILs were characterized for their genotype of the *ELF3* (*J/j-1*/*j-x*), *E1* (*E1/e1-as*), *E2* (*E2/e2*), *E3* (*E3/e3*), and *Dt1* (*Dt1*/*dt1* R166W/*dt1* P113L) genes relevant to their parental genotypes (Additional file 2).

### Days to flower (DTF) and days to maturity (DTM) of five RIL populations

The five RIL populations were evaluated for soybean phenology (DTF and DTM) in low latitude field environments. The RILs, parental lines, and controls were grown for 2 years at 5 locations in northern Ghana (9°N) and days to flower and maturity were determined.

All experimental lines across all RIL populations and environments were analyzed by ANOVA for DTF, DTM, and yield (Additional file 3). Statistical analysis of DTF and DTM validated the precision of the trials; however, the coefficient of variation for yield data was too high to be considered useful (CV = 92%). There were significant differences for each trait due to genotype, environment, and genotype*environment effects (Additional file 3) however in the individual genotype analysis used in this study, genotype*environment interactions were not observed, so the interaction was not explored more (data not shown).

Histograms for RILs of all populations for DTF and DTM demonstrated a skew towards later flowering and maturity more similar to the long juvenile parent rather than the conventional juvenile parent, except for the X97-Jen population (Additional file 4). Although no RILs were earlier flowering or maturing than their conventional parent, there was transgressive segregation for later flowering and maturity than the long juvenile parent.

### In a Jake background, the two variants of the long juvenile trait exhibit different DTF and DTM

**Fig. 1:**
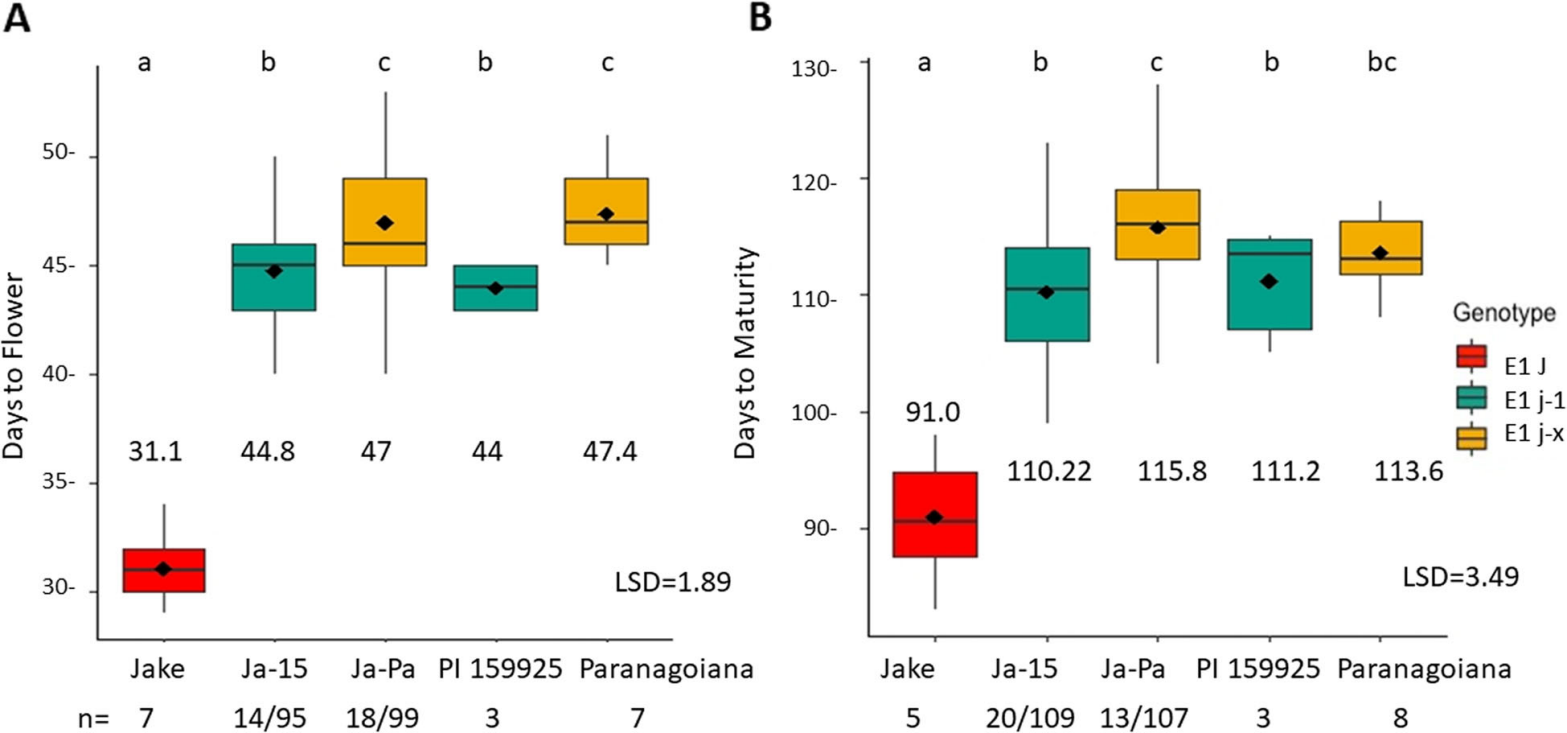
Days to flower and days to maturity in Jake x long juvenile soybean RIL populations grown in 2 years in five locations/2 replications in northern Ghana. For RILs n = number of lines with genotype/ number of site years grown for all genotype. For RILs, n = number of lines with genotype/ number of site years grown for the genotype; for parents, n = site years grown. **a**: DTF for Jake-15 and Jake-Pa and parents. **b**: DTM for Jake-15 and Jake-Pa and parents

To understand the different effects of polymorphisms of *ELF3* on days to flower and maturity, the RIL populations Ja-15 and Ja-Pa were evaluated for different mutant alleles of *ELF3* (*j-1 or j-x*); these RILs had fixed functional alleles of *E1, E2,* and *E3* and were fixed for *dt1* (Table 2). Means for DTF and DTM for RILs and parents were analyzed using Fisher’s LSD (*p* = 0.05) (Fig. 1). When contrasted to RILs with *j-1*, the conventional juvenile parent Jake flowered ~ 14 days earlier. Both the parent Paranagoiana and RILs derived from Paranagoiana with the *j-x* allele showed a significant difference in days to flower compared to the RILs with the PI 159925 *j-1* allele of ~ 2 days (Fig. 1a). The conventional parent Jake reaches maturity ~ 19 days before the Jake-15 RILs containing the *j-1* allele, and RILs with the *j-1* allele mature ~ 5 days before RILs with *j-x* allele from Paranagoiana. However, the parents PI 159925 and Paranagoiana did not show this same difference in days to maturity (Fig. 1b).

### The missense allele of the major maturity gene *E1, e1-as*, influences DTF but does not affect DTM

**Fig. 2:**
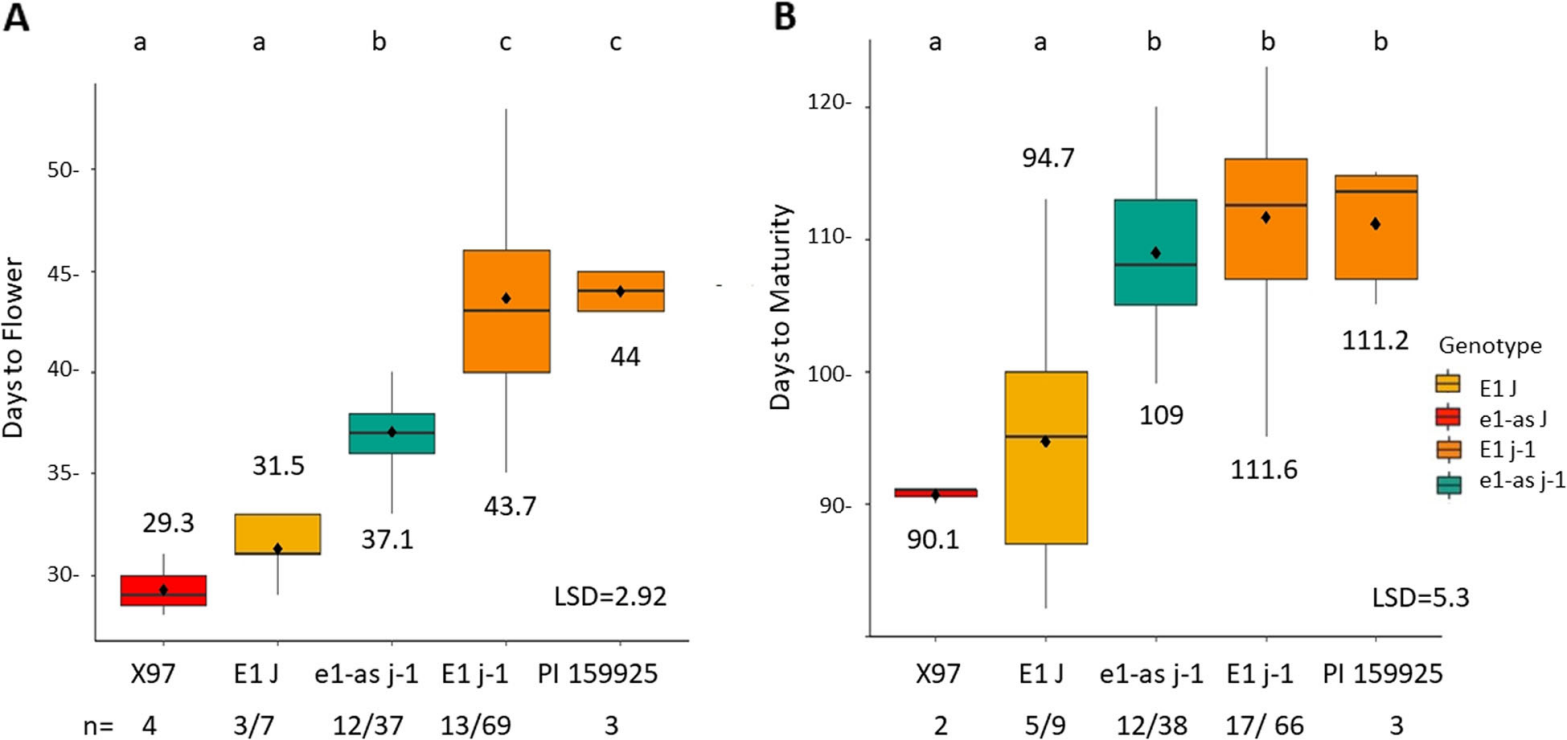
Days to flower and days to maturity in a soybean RIL population that was segregating for *e1-as, E1, J, and j-1*. Parents and RILs were grown for 2 years in five locations/two replications in northern Ghana. For RILs, n = number of lines with genotype/ number of site years grown for the genotype; for parents, n = site years grown. **a**: DTF. **b**: DTM

To test the effect of the allelic combinations of *e1-as*, *E1,* conventional juvenile *ELF3* (*J*) and the PI 159925 long juvenile trait *(j-1),* we utilized the X97–15 RIL population (Table 2). Means of each genotype combination for DTF and DTM were compared (Fig. 2). No RILs were present with the *e1-as*_ *J* genotype in this population. Contrasting the parent X97 with the *e1-as_ J* genotype to RIL lines with the *E1_ J* genotype, there was not a significant difference in DTF between the two genotypic classes. When comparing *e1-as* with the long juvenile trait (*e1-as_j-1*) to *E1* in a conventional juvenile background (*E1_J*) there was a 5.6 difference in DTF when *j-1* was present. Finally, there was a 6.6 DTF difference in *E1* versus *e1-as* in a long juvenile background, which was a similar result as the PI 159925 parent (Fig. 2a). Interestingly, these differences were not seen in days to maturity. The only significant difference was between the genotype groups that were conventional or long juvenile, regardless of the *E1* status, with a difference of ~ 14 days when *j-1* was present (Fig. 2b).

### *E2* affects DTF and DTM in the 534-Can population

**Fig. 3:**
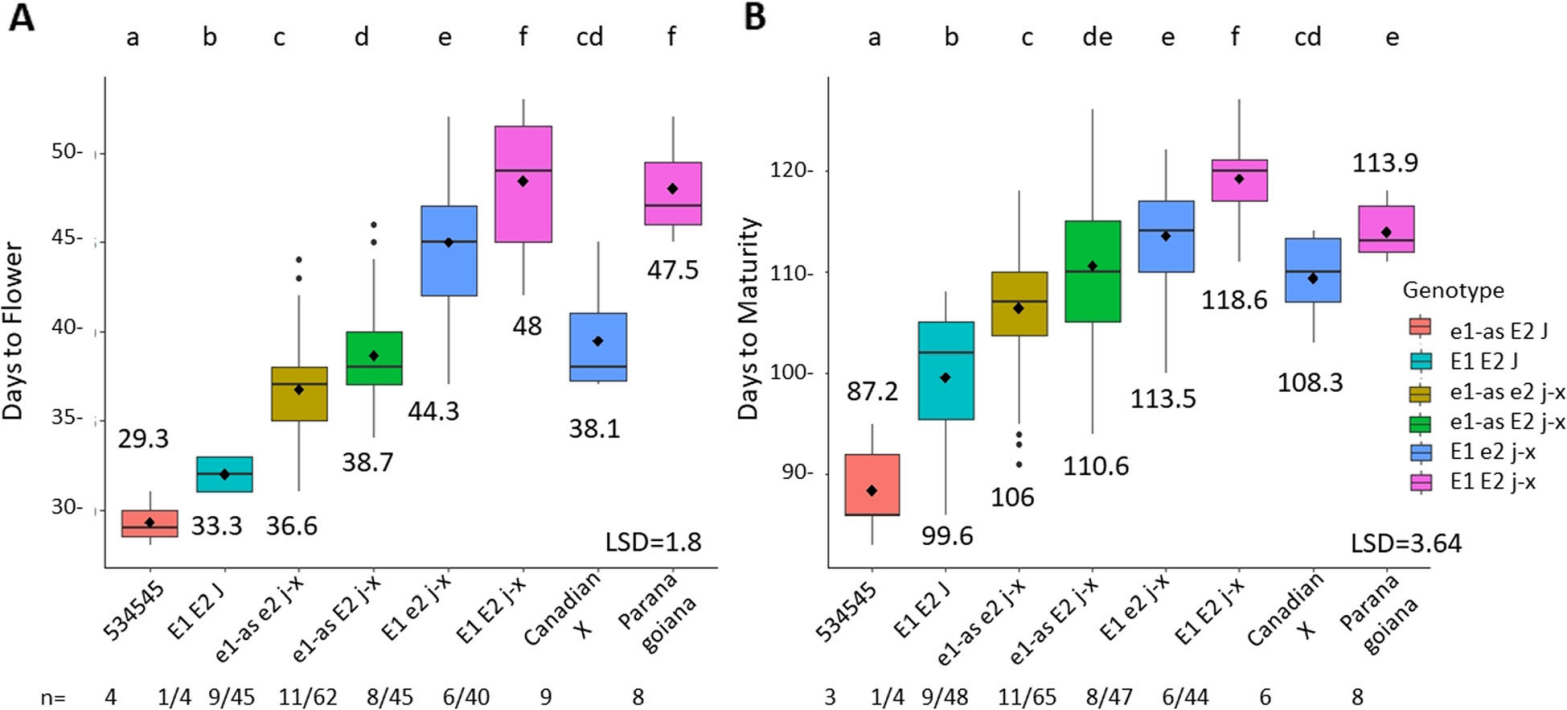
Days to flower and days to maturity in the 534-Can RIL population segregating for *E1/e1-as*, *E2/e2*, *J/j-x*. Data from the individual RILs were analyzed together based on their genotype. Parents and RILs were grown for 2 years in five locations/two replications in northern Ghana. For RILs, n = number of lines with genotype/ number of site years grown for the genotype; for parents, n = site years grown. **a**: DTF. **b**: DTM

To understand how *E2* affects days to flower and days to maturity in tropical environments, a population was created that was segregating for *E1/e1-as, E2/e2, and J/j-x.* The long juvenile donor parent was Can X (*E1_e2_j-x*) and the conventional parent was the food grade soybean 534545 (*e1-as_E2_J*). We categorized DTF and DTM data based on genotype and performed a multiple means comparison test for genotypes from the population. The 534-Can RILs had 5 different genotypes (Fig. 3). There was one conventional genotype group *E1_E2_J* which flowered the earliest at 33 days. All genotype groups significantly increased DTF in a stepwise fashion as alleles that delay flowering were added and all RIL genotype groups were significantly different from each other. The Can X parent (*E1_ e2_ j-x*) had a similar mean to the *e1-as_ j-x* groups, and the long juvenile donor Paranagoiana had similar days to flower as the *E1_E2_j-x* genotypes (Fig. 3a). DTM increased significantly as alleles were added that delay flowering. All genotype groups were significantly different for DTM with the exception of *e1-as_E2_j-x* and *E1_e2_j-x*. The Can X parent (*E1_e2_j-x*) had a similar maturity to the *e1-as_ j-x* genotype groups again. The long juvenile donor Paranagoiana (*E1_E2_j-x*) had a similar maturity to the *E1_e2_ j-x* genotype group. The RIL genotypes *E1_ E2_ j-x* had ~ 5 longer DTM compared to Paranagoiana (Fig. 3b).

### *E2* and *E3* have an additive effect to delay flowering and maturity in an *E1* background in 534-Can

**Fig. 4:**
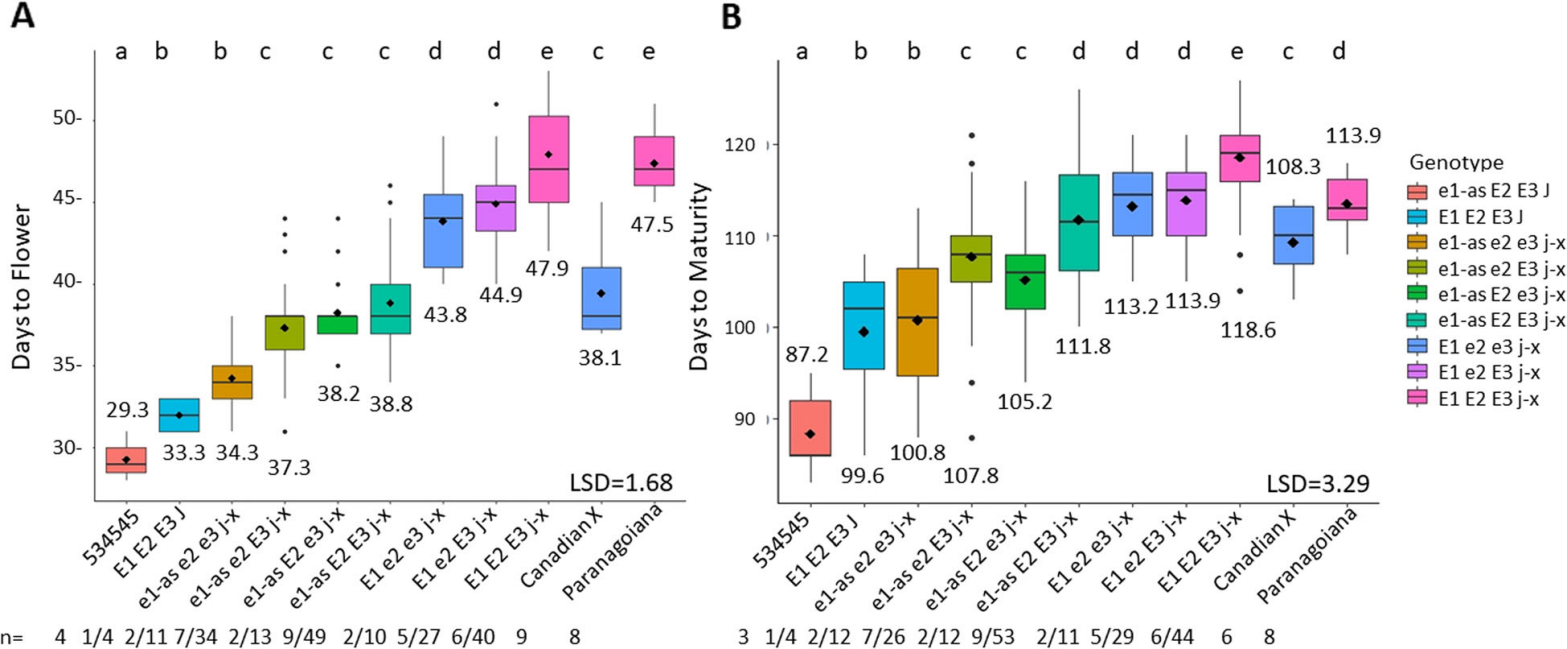
Days to flower and days to maturity in the 534-Can RIL population segregating for *E1/e1-as*, *E2/e2*, *E3/e3*, *J/j-x*. Data from the individual RILs were analyzed together based on their genotype. Parents and RILs were grown for 2 years in five locations/two replications in northern Ghana. For RILs, n = number of lines with genotype/ number of site years grown for the genotype; for parents, n = site years grown. **a**: DTF. **b**: DTM

To dissect the effect of *E3* with *E1, E2,* and *J* allelic combinations, we compared the means of eight different genotypic groups in 534-Can (Fig. 4). There were four significantly different mean DTF groupings that revealed that the effect of *E3* was not consistent across genotypic groups. The *E3* allele status split the *e1-as_ e2_j-x* genotype group into an earlier DTF mean for *e3* RILs and a significantly later DTF mean for *E3* RILs by about 3 days. This was similar to *e1-as_E2_e3 j-x* and *e1-as_E2_E3_j-x*, where although the latter two groups did show delayed flowering as functional alleles were added, the differences were not significant. The next significant difference in days to flower occurred with the addition of *E1* in the *e2 j-x* background, although the presence of *E3* or *e3* did not have a significant effect on DTF. Finally, when all *E* alleles are functional in a *j-x* background, the latest flowering was observed which was also similar to the long juvenile donor Paranagoiana (Fig. 4a). In DTM, the *e3* RILs compared to the *E3* RILs with otherwise identical genotypes showed two cases of significantly different DTM of ~ 7 days earlier when *e3* for both *e1-as*_*e2*_*j-x* and *e1-as_E2*_*j-x*. In the other case, there was no significant difference in DTM observed for *e3* versus *E3* in the groups with *E1_e2_j-x* (Fig. 4b)*.*

### Effects of the *j-1* and *j-x* alleles of the long juvenile trait in different genetic backgrounds

**Fig. 5:**
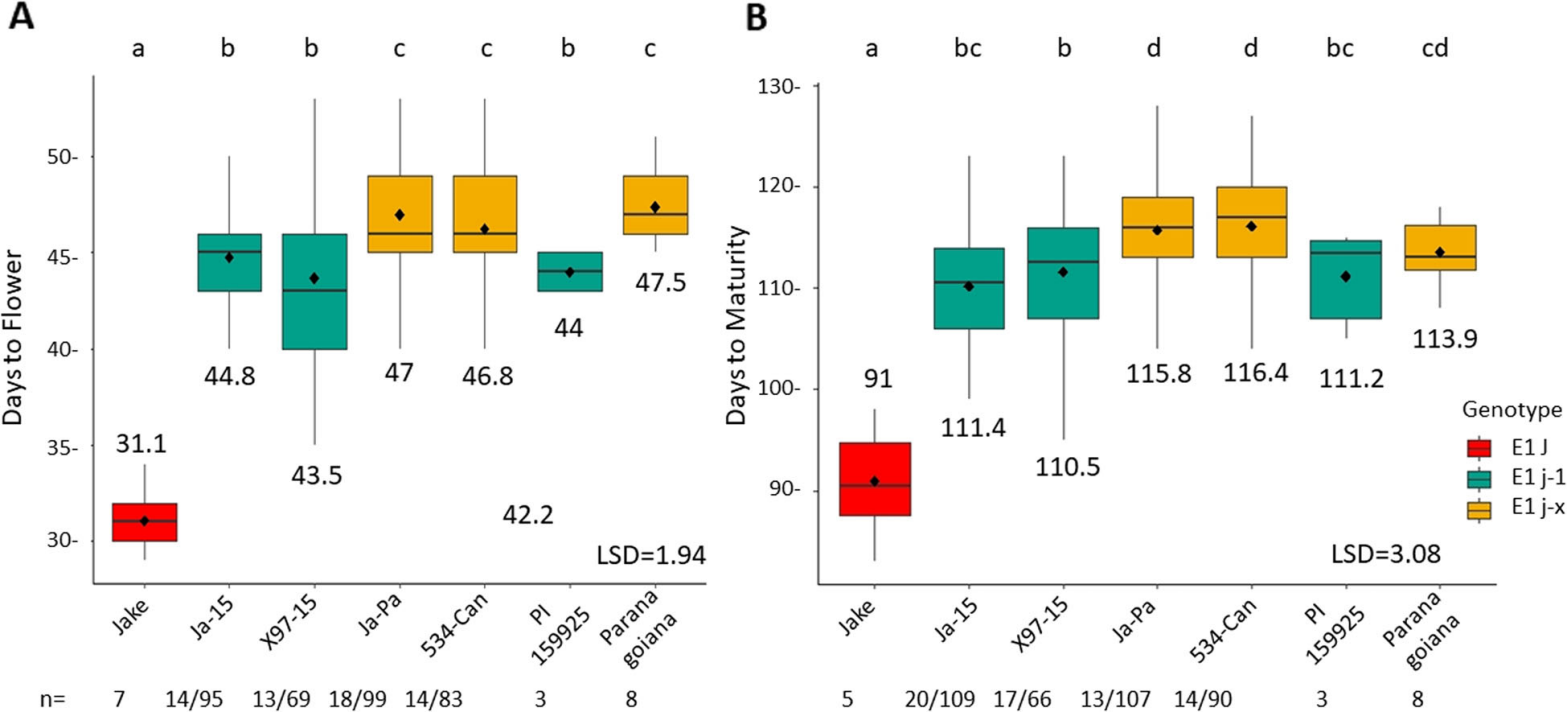
Days to flower and days to maturity for all RILs and parents with a fixed *E1* background. Data from the individual RILs were analyzed together based on their genotype. Parents and RILs were grown for 2 years in five locations/two replications in northern Ghana. For RILs, n = number of lines with genotype/ number of site years grown for the genotype; for parents, n = site years grown. **a**: DTF. **b**: DTM

To confirm that the phenotypes observed in *j-1* and *j-x* alleles are consistent in different genetic backgrounds, we compared DTF and DTM with those alleles when fixed for functional *E1*, but otherwise in different genetic backgrounds. A multiple means comparison test was performed across four RIL populations: Jake-15, X97–15, Jake-Pa, and 534-Can, where the *E1* and *J* genotype of each line was used for grouping within populations. Two populations, Jake-15 and X97–15, were segregating for the *j-1* long juvenile trait allele from PI 159925. There were also two populations segregating for the *j-x* long juvenile allele from Paranagoiana: Jake-Pa, and 534-Can. A comparison was made for DTF and DTM for *E1_ j-1* and *E1*_ *j*-x RILs along with several parent lines (Fig. 5). E1*_ j-1* lines from the X97–15 population were not significantly different for DTF compared to lines in the Jake-15 population with the same genotype or from the long juvenile parent PI 159925. The Jake-Pa and 534-Can RILs with *E1_ j-x* genotype did not show significant difference in DTF, but both *E1_ j-x* genotype groups were significantly later than *E1_ j-1* categories by at least 2 days (Fig. 5a).

In DTM, the Jake-15 and X97–15 lines with *E1_ j-1* did not have a significant difference in DTM between each other or their parent PI 159925 but were significantly later than the conventional parent by ~ 20 days. Jake-Pa and 534-Can with *E1_ j-x* were not significantly different in days to maturity from each other but matured ~ 2 days later than their parent Paranagoiana and were later than *E1_ j-1* by 4–5 days (Fig. 5b).

### The *Dt1* gene influences plant height but not DTM in tropical environments

**Fig. 6:**
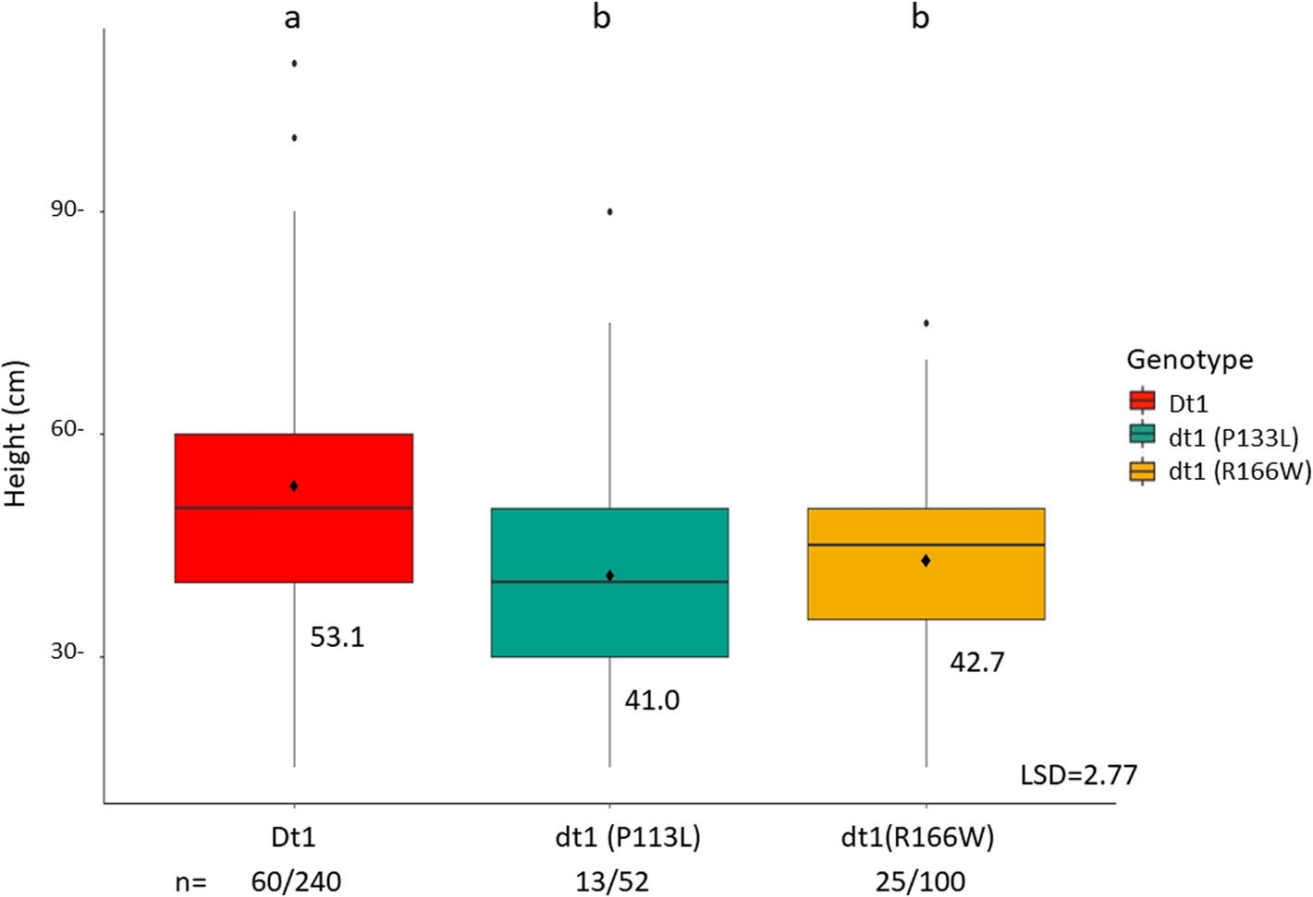
Height data across all populations grouped together based on genotype. Data from the individual RILs were analyzed together based on their genotype. RILs were grown for 2 years in five locations/two replications in northern Ghana. n = number of lines with genotype/ number of site years grown for all genotype

To determine if alleles of the *Dt1* gene have an influence in a tropical environment, plant height was recorded for all populations in 2017. A means comparison was done based on *Dt1* allele regardless of population. There was a significant height difference greater than 10 cm when the indeterminate *Dt1* allele was present compared to determinate lines regardless of the *dt1* allele present (Fig. 6). A means comparison was performed to determine if *Dt1/dt1* had an effect on DTM, but there were no significant differences when lines were long juvenile (data not shown).

## Discussion

Soybean production is expanding to equatorial areas of the world allowing smallholder subsistence farmers access to this economically important crop [5, 42]. Soybean is an invaluable crop for the developing world as it offers resiliency: farmers can choose to sell their seed to livestock feed markets or can directly consume the soybean to benefit from the high protein and calories [1]. However, there are still many obstacles that must be overcome for soybean to be accepted such as accessibility to high quality seed and profitability [43]. Both of these challenges can be met with skilled breeding practices that strive for achieving maximum yields in a low latitude environment. One aspect of breeding soybean in this challenging environment is understanding the genetic mechanisms controlling DTF and DTM, because soybean is a photoperiod sensitive plant that is not adapted to the characteristic 12-h days near the equator, resulting in low yields [20]. Our results can help facilitate further research and development efforts to breed for the correct adaptation to season length to ensure the local farmer has an optimally adapted variety. This research also allows the adoption of new breeding technology that utilizes molecular markers for determining maturity in West Africa.

Our study aimed to understand the role and interactions of *E* genes and alleles of the long juvenile trait by conducting field tests in low latitude West Africa of RILs from five different populations that were segregating for different allelic combinations of our genes of interest. Most importantly, we found that the addition of the long juvenile trait delayed flowering a minimum of 13 days and delayed maturity by 19 days, proving that the long juvenile trait is a critical feature for adaptation to tropical environments [37] (Fig. 1). We found that in a Jake background, the two different alleles of *ELF3*: *j-1* and *j-x* have significantly different DTF and DTM (Fig. 1) with *j-x* being later flowering. In addition, we determined that *E1* and *e1-as* influence DTF but not DTM in a *j-1* background (Fig. 2). These results suggest that it is possible to control soybean season length through the choice of the long juvenile allele, and that the vegetative to reproductive ratio can be adjusted through the selection of *e1-as* or *E1*. Our results are consistent with other studies that show that the *E1 or e1-*as alleles influence different days to flower in a long juvenile background [39]. We suggest that in past studies where a 1:15 segregation ratio for the long juvenile trait was observed, the second gene was most likely *E1* [36, 38].

The 534-Can population of RILs with *j-x* showed a stepwise increase in days to flower and maturity as functional *E* alleles were added. 534-Can RILs experienced significant delays in flowering and maturity when functional alleles of *E2* or *E3* were present (Figs. 3-4). This is in contrast to the *j-1* allele which does not show a significant difference in maturity even when alleles of *E1* are contrasting (Fig. 2). Further population development and testing will need to be done to understand if other alleles of *E* genes are capable of affecting maturity in a *j-1* background. Our results point to a hierarchy of effects for DTF and DTM where *j* > *E1* > *E2* > *E3* in low latitude environments.

The lines used in this study were RILs from five different populations that interrogated the effects of the maturity genes in a variety of genetic backgrounds. To ensure that these results can translate into useful breeding information, the same allele combinations were investigated in different genetic backgrounds (Fig. 5). We looked at results of two genotypes: *E1_j-1* and *E1_j-x* in two different backgrounds each. For *E1_j-1* there were 14 lines from the Ja-15 population and 13 lines in the X97–15 population. For *E1_j-x* there were 18 lines from the Ja-Pa population and 14 lines from the 534-Can population. The results remained consistent regardless of the genetic background, where no significant difference was observed within similar genotypes in different backgrounds, but the same significant difference is seen between *E1_j-1* and *E1_j-x* in different backgrounds. This suggests the maturity genes *E1* and *J* are critical to breeding efforts in West Africa, and if used in other genetic backgrounds, similar results can be expected.

It is also important to note that there is also natural selection against unadapted varieties. There were a very low number of RILs with the genotype *e1-as_J* or *E1_J* that survived to produce sufficient seed for additional generations both during population advancement in Costa Rica and during trials in Ghana. Pod shatter is also devastating to yields, and the gene controlling a large percentage of the shatter phenotype, *Pdh1* [44], was present in populations with PI 159925 as a parent. While all of the populations had a reduction in tested RILs from 2016 to 2017, the two with PI 159925 and thus *Pdh1* segregating had the largest reductions in RILs. There seemed to be natural selection for functional alleles of the *E* genes and the long juvenile trait based on the number of lines that survived (Additional file 4). Natural selection for delayed flowering and maturity was especially apparent in the 534-Can population where many of the RILs were tested with the *E1_E2_E3_j-x* genotypes.

The long juvenile genetic mechanism in most African varieties including Jenguma is not known, although research in *J* varieties has produced new potential candidate genes besides *ELF3* [45]. There is a possibility that breeding with *j* alleles of *ELF3* could have yield benefits through optimization of season length and vegetative to reproductive stage ratio, although this would need to be evaluated in a field setting. Our research has shown it is possible to manipulate the vegetative to reproductive stage ratio through the *E1* allele chosen in a *j-1* background, and it may be possible to add finer regulation of DTF and DTM with *E2* and *E3* alleles in a *j-x* background. This knowledge and these alleles should be implemented in West African breeding programs as is needed in certain tropical environments, and tested for possible yield benefits.

Taken together, it is possible to control tropical soybean time to maturity through the selection of long juvenile alleles and also the DTF through selection of *E1* or *e1-as*, and possibly *E2* and *E3* in certain backgrounds. As has been mentioned in previous studies, there are still background effects that influence long juvenile trait maturity phenotypes [18]. This research will allow soybean breeders to evaluate the impact on yield by consciously manipulating season length and the vegetative to reproductive ratio.

## Conclusions

Here we present low latitude field analysis conducted in northern Ghana of two alleles of the *ELF3* long juvenile trait, *j-1* and *j-x* in combination with functional or nonfunctional alleles of *E1*, *E2*, and *E3*. We have shown that there are significant differences in DTF and DTM in different allelic combinations including *j-x* has more delayed DTF and DTM than *j-1*. Alleles of *E1* influence DTF but not DTM in a *j-1* background. Alleles of *E1* and *E2* influence DTF and DTM in a *j-x* background, and *E3* may also have a slight effect. Alleles of *Dt1* influence plant height but not maturity. Further research needs to be done to understand how these allelic combinations affect yield in a low latitude environment.

## Methods

### Plant materials

Seven soybean parents were chosen to create experimental populations (Table 1). Five recombinant inbred line (RIL) populations were created for this study, where each had one conventional juvenile parent (*J*) and one long juvenile parent (*j*) (Table 2). Three conventional parents were utilized. Jake is a high yielding MG V determinate American variety released by the University of Missouri [46]. X97–0101 (referred to as X97 for the duration of this paper) is a lectin-free, trypsin inhibitor-free isogenic experimental derivation of indeterminate MG III Williams 82 developed by the University of Illinois [47]. 534545 is an indeterminate MG III food grade soybean variety, utilized for its high protein and sucrose content shared by the Missouri Soybean Merchandising Council [48]. Four long juvenile parents were utilized. PI 159925 is a determinate plant introduction line from Peru which was obtained from the soybean germplasm collection at Champaign-Urbana, Illinois (https://www.ars-grin.gov/ npgs/ index.html). It was the first line in which the long juvenile trait was characterized, and the allele is designated *j-1* [18, 39]. PI 159925 is the only parent utilized in this study with the shatter susceptible alleles of *Pdh1* [49, 50]. Paranagoiana (PI 628880) was identified from natural variation in the Brazilian released determinate variety Paraná (PI 628879) that contains the long juvenile trait designated herein as *j-x* [37, 41]. This line was also obtained from the soybean germplasm collection at Champaign-Urbana, Illinois (https://www.ars-grin.gov/ npgs/ index.html). X5683-1-18 (referred to as Can X for the duration of the paper) is an experimental indeterminate backcross 5-derived line created by using the early maturing OT94–47 as a recurrent parent and Paranagoiana as the long juvenile *j-x* donor developed by Agriculture and Agri-Food Canada [38]. Jenguma is a released soybean variety developed and provided by the Savanna Agricultural Research Institute for production in Ghana. All soybean materials were obtained with permission.

### RIL populations and field experimental design

The soybean populations (Table 2) originated from crosses made at the South Farm Research Center near Columbia, MO (SF) in summer 2013 (Jake-15 and Jake-Pa) or 2014 (X97-Jen and 534-Can X), or in Upala, Costa Rica (10.8979°N, 85.0155°W) in January of 2014 (X97–15). The F_1_ seeds for the Jake-15, Jake-Pa, and X97–15 populations were grown and self-pollinated to produce F_2_ seeds at SF in summer 2014. The F_1_ seeds for X97-Jen population were self-pollinated to produce F_2_ seeds in February 2015 in Upala, Costa Rica. The F_1_ seeds for the 534-Can X population were self-pollinated to produce F_2_ seeds in January 2015, then advanced another generation to produce F_3_ seeds in May 2015 all in Upala, Costa Rica.

The Jake-15, Jake-Pa, and X97–15 F_2_ populations were grown in Upala, Costa Rica for plant development phenotyping from December 2014 through April of 2015. One hundred seeds of each line were planted in a single row per population, and at 81 days after planting, individual F_2_ plants were evaluated for plant development stage. There were 92 F_2_ plants in each of the Jake-15 and Jake-Pa populations, and 90 F_2_ plants in the X97–15 F_2_ population. In the Jake-15 and Jake-Pa populations, plants exhibiting long juvenile characteristics (exhibited delayed flowering in a 1:3 ratio) were tagged (21 and 20, respectively), leaf samples were collected on FTA cards for genotyping, and single plant threshes of F_2:3_ seeds of each of the long juvenile plants were made after the plants matured. All of the X97–15 F_2_ population plants were single plant threshed.

In May of 2015, population development for recombinant inbred lines (RIL) by single seed descent to F_4:6_ bulks was initiated in Upala, Costa Rica for all populations using unselected F_2:3_ seeds (X97–15 population), unselected F_3_ seeds (534-Can X population), and a single F_3_ plant from each of the selected long juvenile F_2:3_ plants from the Jake-15 and Jake-Pa populations. For the X97-Jen population, the RILs were F_3:5_ bulks because the available starting materials were F_2_ seeds in May 2015 in Upala, Costa Rica. Although 100 RILs were targeted for each of the unselected RIL populations, many lines were lost during advancement or did not produce sufficient seed due to inappropriate maturity, seed shatter, or other issues at the bulk stage (April/May 2016 in Upala, Costa Rica). The F_4:6_ RIL (F_3:5_ for X97- Jen) seed for all populations was shipped to Tamale, Ghana in spring 2016 (Additional file 1).

Yields trials were conducted in five fields throughout northern Ghana in 2016 and 2017. The fields were either a Savannah Agricultural Research Institute research field (Nyankpala SARI [NyS, 9.403°N,-1.008°W], Yendi SARI [YeS, 9.495°N,0.128°W], and Wa SARI [WaS, 9.799°N, −2.499°W] or a local farmer’s field (Nyankpala Farmer [NyF, 9.396°N,-1.019°W] and Yendi Farmer [YeF, 9.412°N,-0.102°W]). Planting date was determined by the start of continuous seasonal rainfall and field conditions/availability. In 2016 soybeans were planted on 9 and 11 July in YeF, 13 July in NyF, 15 July in NyS, 16 July in YeS, and 20 July in WaS. In 2016, the YeF maturity and yield data were not collected due to soybean sudden death syndrome devastation. The experimental design was a single experimental line bordered by the local variety Jenguma in randomized complete block design with two replications, where one row of a RIL was bordered by a local check (Jenguma) on both sides. In 2016, blocking was done by population. All rows were hand planted 75 cm apart per IITA’s recommendation (www.iita.org). Plots were ~ 300 cm (10 ft) long with a ~ 122 cm (4 ft) alley above. Granular inoculant was used and applied directly to open furloughs immediately before seeds were planted and covered. No fertilizer was used to represent local farmer practices and to replicate farmer agronomic and yield results. In 2016, 120 seeds were planted in each plot to compensate for predicted poor germination. Glyphosate was sprayed after planting and before emergence. Weed control was manual after emergence. Plots exceeding 100 plants per row were thinned to 100 during emergence note taking. Flowering date (R1) was determined when 2+ plants had opened flowers in the center of a plot to eliminate environmental influence on individual plants on plot ends. Plots were considered mature when 95% of pods were dried (R8) [51]. Height from the ground to the apical meristem of random individuals in each plot was taken immediately before harvest. Harvest was done by hand and threshed mechanically using an Almaco single bundle thresher. Seeds were cleaned using sieves and by hand picking and then weighed for yield. Seed yield was calculated as grams per 10-ft row. YeS and NyF produced the highest quality seed and was stored in a 4 °C cold room for planting in 2017.

The 2017 field and experimental design was identical to 2016 with some exceptions. Lines were eliminated from field tests in 2017 if they did not produce enough seed to be planted in 5 locations or if they exhibited a segregating phenotype in 2016. Populations that had PI 159925 as a parent suffered yield losses due to shatter. PI 159925 contains the *Pdh1* [44] shatter-prone allele. The X97–15 population experienced heavy seed loss in Ghana, where the population size for the multi-location field test was reduced from 47 RIL in 2016 to lines to 5 in 2017 due to insufficient seed produced by the other 42 lines. In Jake-15, only 9 RILs of 20 were tested in 2017 due to low seed production. In 2017, fields were planted 8 July in YeF, 10 July in YeS, 11 July in NyF, 18 July in NyS (replanted 2 August), and 21 July in WaS.

Two hundred seeds were planted per plot for to compensate for predicted low germination. In 2017, NyS no data were collected due to flooding damage that resulted in poor emergence.

The daylength in July in northern Ghana is 12.4 h and the daylength in December is 11.4 h (www.timeanddate.com).

### Genotyping

#### DNA extraction

Initial genotyping was done with leaf presses on FTA cards (Whatman, Clifton, NJ) taken in Ghana in 2016 from trifoliates in R1 and shipped to Columbia, Missouri as described in [52]. Missing data was genotyped again in 2017 in Columbia, Missouri using F_7_ seed that was shipped from Ghana. DNA was extracted from 2 to 5 seeds using the DNeasy Plant Mini Kit (Qiagen, Valencia, CA) and followed the protocol described in [23].

#### E gene genotyping assays

*E1* and *E2* genotyping assays were conducted as described in [23]. *E3* genotyping assay was conducted as described in [22].

#### Dt1 genotyping assays

##### *Dt1/ dt1* R166W

All SimpleProbe assays described are similarly developed as in [50]. A SimpleProbe melting curve assay was developed to determine the adenine to thymine *dt1 R166W* missense allele from the wildtype *Dt1* (Glyma.19G194300, Wm82.a2.v1). The primers Dt1in31f (5′-CATGAGAGAGATCACTGAC-3′) and Dt1endr1 (5′- GCAAAACCAGCAGCTACTT-3′) amplify a 292-bp region, which includes the T/A SNP at position 45,183,701 in *Glycine max* Wm82.a2.v1 genome. The SimpleProbe oligonucleotide (5′- Fluorescein-SPC-TGCACAGAGGGAAACGGCT-Phosphate −3′) was designed using the LightCycler Probe Design software (Roche Applied Science, Indianapolis, IN) and anneals to the sense strand. PCR reactions were 20 μl and included the DNA template, 0.5 μM reverse primer Dt1endr1, 0.2 μM forward primer Dt1in31f, 0.2 μM SimpleProbe, buffer (40 mM Tricine- KOH [pH 8.0], 16 mM MgCl_2_, 3.75 μg ml^− 1^ BSA), 5% DMSO, 200 μM dNTPs, and 0.2X Titanium Taq polymerase (BD Biosciences, Palo Alto, CA). PCR reactions were run on the LightCycler 480 real-time PCR instrument (Roche Applied Science, Indianapolis, IN). Reactions were denatured at 95 °C for 3 min, and then in each cycle denatured at 95 °C for 20 s, primers annealed at 60 °C for 20 s, and products elongated at 72 °C for 20 s for 45 cycles. After amplification was completed, a melting curve was conducted from 50 to 70 °C. The *dt1 R166W* mutant allele peak was observed at 57 °C, and the *Dt1* wildtype peak was observed at 63 °C. Heterozygous *Dt1/dt1* samples produced both peaks.

##### Dt1/dt1 P113L

For detection of the P113L missense *dt1* alleles, a cleaved amplified polymorphic sequence assay was developed based on the introduction of a *HindIII* restriction enzyme site in the P113L *dt1* alleles at position 45,183,859 (G/A) in *Glycine max* Wm82.a2.v1 genome [53]. PCR products of 292 bp were amplified in 20 μl reactions containing DNA template with Dt1in31f and Dt1endr1 primers (as above) at 0.5 μM and buffer (40 mM Tricine- KOH [pH 8.0], 16 mM MgCl_2_, 3.75 μg ml^− 1^ BSA), 5% DMSO, 200 μM dNTPs, and 0.2X Titanium Taq polymerase (BD Biosciences, Palo Alto, CA). Reactions were denatured at 95 °C for 3 min, and then in each cycle denatured at 95 °C for 20 s, primers annealed at 60 °C for 20 s, and products elongated at 72 °C for 20 s for 45 cycles. After amplification was completed 5 μl of each sample was removed to check for product formation on the FlashGel system (Lonza, Basel, Switzerland). To the remaining 15 μl of each sample, an enzyme mixture (15 μl) was added that contained 1.5 μl New England BioLabs (NEB, Ipswich, MA) buffer 2, 1.5 μl NEB *HindIII* (30,000 units), and 12 μl of ddH_2_0. Reactions were incubated overnight at 37 °C, and products were separated on the FlashGel system. The *Dt1* genotype produced a 215 bp band, while *dt1* P113L genotypes produced bands of 215 bp and 77 bp, and heterozygous samples produced bands of 292, 215, and 77 bp.

#### ELF3 genotyping assays

##### *j-1*: cytosine deletion (C-del) found in PI 159925

For detection of the long juvenile trait C-del in the PI 159925 version of *ELF3 (*Glyma.04G050200, Wm82.a2.v1), a SimpleProbe assay was created. The primers Cdelfor (5′-TGTTCTGCAGAGAATGCGGT-3′) and Cdelr (5′- CCTCCTCCACAACCAGTTCC-3′) produce a 254-bp PCR product that contains the C/− SNP described at position 4,077,102 (Lu et al. 2017). The SimpleProbe oligonucleotide (5′-Fluorescein-SPC-GACGGTAGCCACCTTTCAAAATGCA-Phosphate-3′) was designed on the sense strand using the LightCycler Probe Design software (Roche Applied Science, Indianapolis, IN). PCR was identical as the *Dt1/dt1 R166W* assay with the exception that the melting curve was from 50 to 75 °C. The C-del mutant allele peak was observed at 61 °C, and the *ELF3* wildtype peak was observed at 68 °C. Heterozygous samples produced both peaks.

##### *j-x*: unknown mutation in Paranagoiana

The exact polymorphism controlling the long juvenile trait in Paranagoiana is not known, but it is believed to be tightly associated with *ELF3* [41]. Sequencing of *ELF3* DNA from Paranagoiana also did not produce any polymorphisms except for our difficultly to amplify and sequence the junction between intron 3 and exon 4. To distinguish the Paranagoiana *j-x* alleles, we developed a gel-based assay with PCR targeting both *ELF3* and a control region on another chromosome to ensure PCR was successful. The primers ljkf. (5′- CGAGTATTGTGCAATTTTCTTGATCC-3′) and Cdelr: (5′- CCTCCTCCACAACCAGTTCC-3′) amplify a 652-bp region that includes the intron 3 to exon 4 junction. The control primer set lx1f (5′- ACCGACATCTTAGCGTGCTT-3′) and lx1r (5′-AAAAAGGTTGTCTCTATTATGCCAT-3′) amplifies a 129-bp region of the lipoxygenase gene on chromosome 13 (Glyma.13G347600).

PCR reactions were 20 μl and included the DNA template (this assay did not work with DNA from leaf presses), 0.5 μM *ELF3* reverse primer Cdelr, 0.5 μM *ELF3* forward primer ljkf, control primers: 0.25 μM lx1f and 0.25 μM lx1r, buffer (40 mM Tricine- KOH [pH 8.0], 16 mM MgCl_2_, 3.75 μg ml^− 1^ BSA), 5% DMSO, 200 μM dNTPs, and 0.2X Titanium Taq polymerase (BD Biosciences, Palo Alto, CA). PCR reactions were run on a thermocycler and were denatured at 95 °C for 3 min, and then in each cycle denatured at 95 °C for 20 s, primers annealed at 60 °C for 20 s, and products elongated at 72 °C for 60 s for 45 cycles. After amplification was completed, PCR products were run on a 1.5% agarose gel containing SYBR Safe DNA gel stain diluted 1:10,000 at (145 V) for 20 min. Products were visualized using a blue-light transilluminator. Only lines that produced product 129 bp for the lx1 primers were assigned a genotype for *J*. If an upper band was present such as in the *J* control, the line was considered conventional, if no 652 bp band was present, it was considered *j-x*.

### Statistical analysis

Days to flower notes were taken three times a week in the Nyankpala fields, once per week in the Yendi fields, and once per week in the Wa field on average in 2016. Days to flower 2017, and days to maturity: 2016 and 2017 were recorded twice per week in Nyankpala fields, twice per week in Yendi, and once per week in Wa. ANOVAs for all data collected were analyzed using PROC GLM procedure in SAS software version 9.4 (SAS Institute. 2012. The SAS 9.4 system for Windows. SAS Inst., Cary, NC). Days to flower data from Wa were not used.

Days to flower, days to maturity, and yield data from all lines were analyzed by ANOVA using the model equation name + environment + rep (environment) + name*environment (Additional file 3). Environment was defined as site years or in other words location_year. There were 9 environments with data recorded.

Data from lines containing the same genotype were grouped together and analyzed by ANOVA as genotype + environment + rep (environment), and genotype*environment. Outliers from each genotype group were removed only after verifying that they were due to note taking error. Data from lines with incomplete genotype data (either missing or heterozygous for at least one gene) were omitted from analysis. After data was cleaned based on these standards, Fisher’s least significant differences (LSDs) were generated using SAS software 9.4 where *p* = 0.05. Boxplots were constructed using the ggplot2 package in R version 3.6.0 [54].

## Supplementary information

**Additional file 1.** Recombinant inbred line population development. A generation by generation plan of plants for all five RIL populations used in this study.

**Additional file 2 **Data for RILS in all populations. Data for RILs including population name, experimental name, 2 yr mean DTF and DTM, genotype for *E1*, *E2*, *E3*, *Dt1*, *J.*

**Additional file 3.** ANOVA tables for DTF, DTM, and yield for all RILS. Three ANOVA for agronomic traits of interest.

**Additional file 4 **Histograms of agronomic traits from five RIL populations. Number of RILs is on the y-axis and days is shown on the x-axis. Data for parents of each population are shown with an arrow with the first letter of the parent name to the right. a: Days to Flower of Jake-15 b: Days to Flower of Jake-Pa c: Days to Maturity of Jake-15 d: Days to Maturity Jake-Pa. a-d: Both populations were selected for the long juvenile trait. e: Days to Flower of X97–15 f: Days to Flower of X97-Jen g: Days to Maturity of X97–15 h: Days to Maturity of X97-Jen e-h: Both populations were segregating for *E1/e1-as* and different alleles of *J/j.* i: Days to Flower of 534-Can j: Days to Maturity of 534-Can. i-j: This population was segregating to *E1/e1-as, E2/e2, E3,e3* or *J/j-x*.

## Data Availability

The datasets used and/or analysed during the current study available from the corresponding author on reasonable request.

## Abbreviations

DTF: Days to flower
DTM: Days to maturity
LSD: Least significant difference
PI: Plant introduction
RIL: Recombinant inbred line
