# Supplementary figures and images for: The effects and interaction of soybean maturity gene alleles controlling flowering time, maturity, and adaptation in tropical environments
Source: BMC Plant Biol. 2020 Feb 7;20:65. doi: 10.1186/s12870-020-2276-y (PMC7006184; doi:10.1186/s12870-020-2276-y)

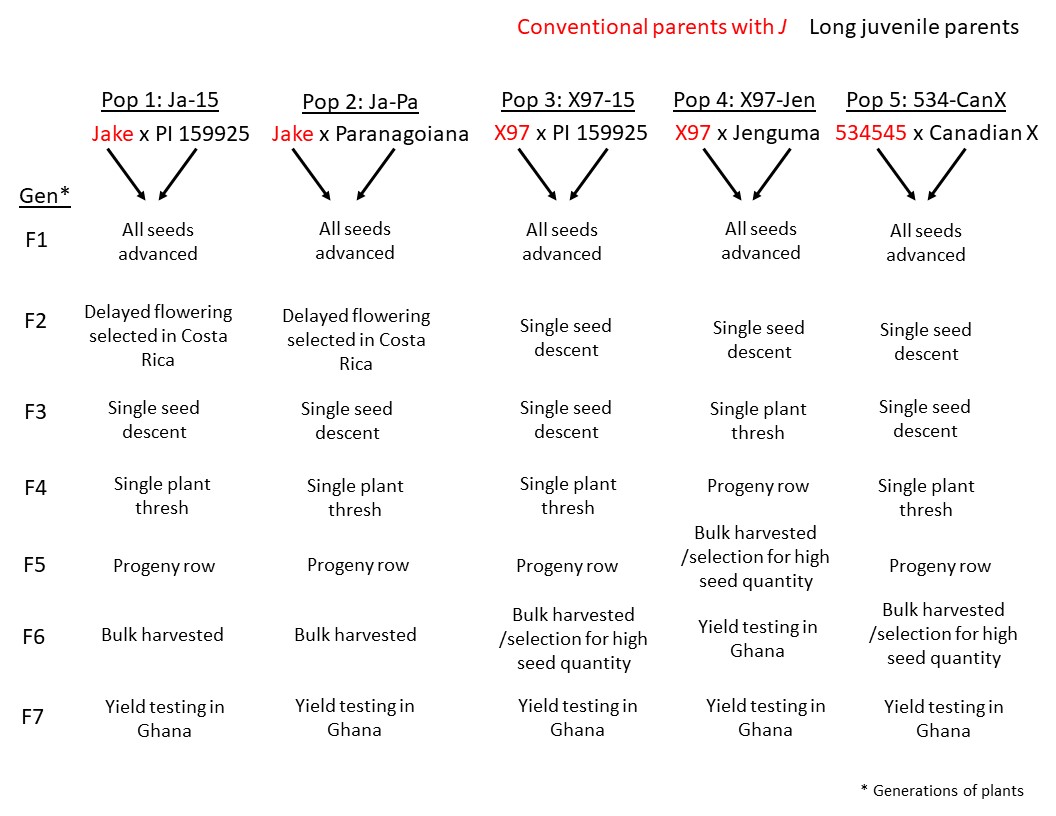

Supplement: Supplementary file 1 — Additional file 1. Recombinant inbred line population development. A generation by generation plan of plants for all five RIL populations used in this study. [file 12870_2020_2276_MOESM1_ESM.jpg]

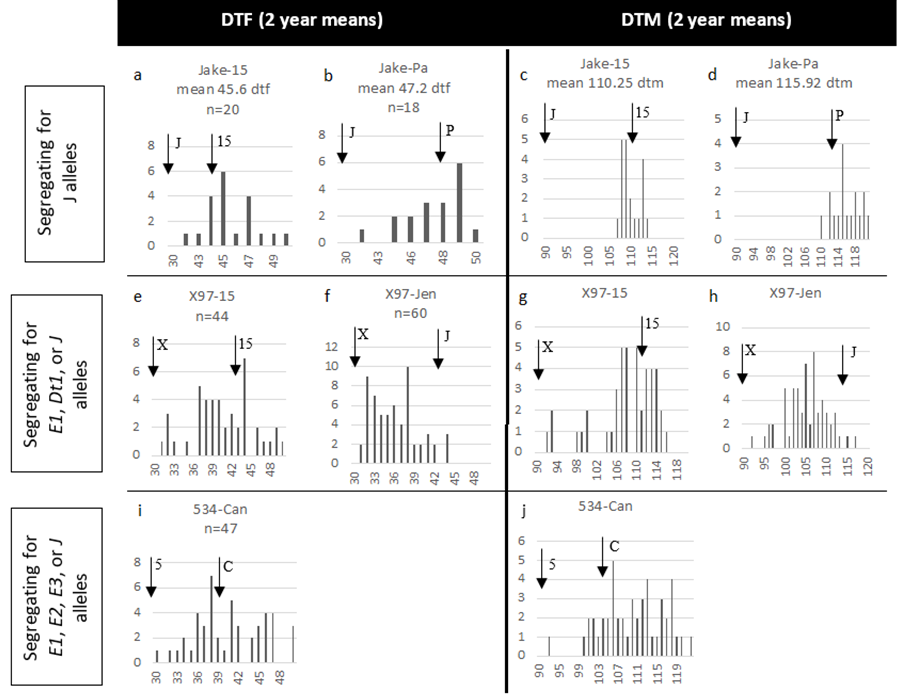

Supplement: Supplementary file 4 — Additional file 4 Histograms of agronomic traits from five RIL populations. Number of RILs is on the y-axis and days is shown on the x-axis. Data for parents of each population are shown with an arrow with the first letter of the parent name to the right. a: Days to Flower of Jake-15 b: Days to Flower of Jake-Pa c: Days to Maturity of Jake-15 d: Days to Maturity Jake-Pa. a-d: Both populations were selected for the long juvenile trait. e: Days to Flower of X97–15 f: Days to Flower of X97-Jen g: Days to Maturity of X97–15 h: Days to Maturity of X97-Jen e-h: Both populations were segregating for E1/e1-as and different alleles of J/j. i: Days to Flower of 534-Can j: Days to Maturity of 534-Can. i-j: This population was segregating to E1/e1-as, E2/e2, E3,e3 or J/j-x. [file 12870_2020_2276_MOESM4_ESM.tiff]
